# Macrophage invasion: here, there and everywhere

**DOI:** 10.1038/s41392-022-01074-z

**Published:** 2022-07-09

**Authors:** Lola C. Hernandez, Pablo J. Sáez

**Affiliations:** Cell Communication and Migration Laboratory, Institute of Biochemistry and Molecular Cell Biology, Center for Experimental Medicine, University Medical Center Hamburg-Eppendorf, Hamburg, Germany

**Keywords:** Innate immune cells, Cell biology

Recently *Akhmanova* et al. reported in *Science* that cell division at specific locations in the tissue constitutes a novel strategy for macrophage infiltration.^1^

Cell infiltration or tissue invasion is a common migratory strategy used by highly motile cells. This cellular response is observed in tissue colonization during development, drives intra/extravasation of leukocytes during the immune response and is exploited by tumors for metastasis. Migrating cells employ diverse mechanisms to invade tissues, including degradation or deformation of the extracellular matrix (ECM) and cell squeezing through small pores.^2^ Leukocytes are professional migrating cells able to find their way through tissues with different topologies and levels of confinement. Using their nucleus, the largest and stiffest organelle in a cell, leukocytes probe the microenvironment and choose the path of least resistance when moving through small pores.^3^ However, how are these paths generated when the tissue is densely packed?

**Fig. 1:**
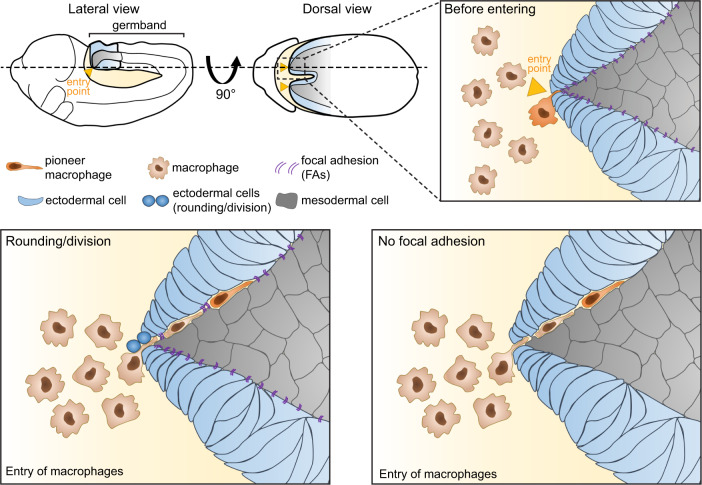
Schematic illustration during macrophage invasion of the germband. Lateral and dorsal view of the *Drosophila melanogaster* embryo. The focal plane of the germband invasion (dashed line) is shown. The entry points (orange arrow) located at the junction of the ectoderm (cyan) and the mesoderm (gray) are depicted. Before invading the germband macrophages accumulate in front of the entry point. Upon rounding during ectodermal cell division at the entry point, the pioneer macrophage enters the tissue. In addition, lack of focal adhesions in the ectodermal layer permits macrophage invasion in absence of rounding/division

Using the embryo of fruit fly (*Drosophila melanogaster*) and in vivo time-lapse microscopy *Akhmanova* et al. studied the spreading process of *Drosophila*’s macrophages (hemocytes or plasmocytes), which are highly migratory cells. Hemocyte spreading is a conserved response that follows the same pattern during development, making it an ideal model to study tissue invasion. Following chemical guidance, macrophages follow pre-determined routes traversing tissues in order to populate the whole embryo. This is a process that recapitulates the establishment of tissue resident macrophage niche in other models. One key step driving this process is the germband invasion by macrophages migrating from the embryo’s head. In order to migrate to the designated tissues, macrophages have to squeeze in between two closely apposed cell layers, the ectoderm and mesoderm (Fig. 1). Despite virtually no space is present between these tissues for cells to sneak in, macrophages penetrate the germband. Interestingly, this process does not require degradation of the ECM, but rather the opposite: in order to migrate efficiently in this tightly knit tissue, macrophages release laminins, a component of the ECM.^4^ This is relevant because having more ECM results in a more complex microenvironment increasing the difficulty of migrating on it. Additionally, part of the same puzzle, upon arrival at the entry point, in the junction of the mesoderm surface and the basal side of the ectoderm, a pioneer macrophage patiently waits before invading the germband, and make room for the following macrophages. Invasion and directional migration are all about being at the right time at the right place, but why does this pioneer macrophage needs to wait? How does this macrophage overcome the physical impediment of the germband cellular wall to follow through?

Highlighting the power of live imaging, *Akhmanova et al* observed that pioneer macrophage invasion coincides with mitosis of one ectodermal cell and not a mesodermal one, creating a gap at the tissue junction. This migratory response happens symmetrically in the embryo, at both sides of the hindgut where two entry points are established (Fig. 1). Macrophages arrive at these two locations almost simultaneously, but they do not enter at the same time if ectodermal divisions at both entry points occur asynchronously. The latter emphasizes that is the division of an ectodermal cell at the entry point what dictates macrophages invasion. By using pharmacological and genetic approaches to prevent or increase ectodermal mitosis, the authors elegantly show that if mitosis is prevented, macrophages are unable to invade the germband. Conversely when mitosis is accelerated macrophages infiltration happens at a faster rate. The contribution of other cells was excluded because the regulation of ectodermal cell mitosis did not severely impair macrophage’s arrival at the entry point nor their migration, nor the overall development of the embryo.

Additionally, the authors found that the ectodermal and mesodermal layer is tightly sealed by integrin-rich focal adhesions (FA) from the basal side of the ectoderm, which anchors laminin filaments of the ECM. Importantly, the sole rounding of the ectodermal cells is not enough to disrupt the physical barrier and allow macrophage entry, because the increase in the gap between the two layers is not enough to allow the passage of a macrophage’s nucleus. The authors show that germband invasion by macrophages requires FA disassembly by the ectodermal cells at the entry point. This process leaves enough space to allow the nucleus of the pioneer macrophage to squeeze through. The disassembly of FAs did not affect the embryo development revealing that macrophage invasion of the germband depends solely on local interactions with the tissue.

The pioneer macrophage only invades the germband when the gap is big enough to allow the passage of its nucleus. This is a relevant fact as it corroborates in vivo some migratory features found in other leukocytes.^3^ In addition, although in this model the opening of small gaps was not enough to allow macrophage infiltration it is likely that small changes in confinement could trigger mechanosignaling pathways contributing to a more efficient invasion.

The authors have previously shown that TNF reduces tissue tension and increase cell division.^5^ In the present work the authors discard an effect of macrophages in triggering ectodermal cell division. However, it is tempting to hypothesize that in other models macrophages could release signals to locally trigger mitosis allowing their invasion.

Another interesting aspect is that macrophages invade the germband in a stream once the pioneer macrophage has entered. Is this collective invasion of individual migrating cells? Which autocrine and paracrine signals coordinate this response? Since cell-cell communication is also key in the maintenance of monolayers, it is likely that the first ectodermal cell signals to the neighbors allowing the passage of the follower macrophages while maintaining FAs intact.

The current paper by *Akhmanova* et al. proposes a novel concept, which could be expanded to a plethora of unexplored models. Does endothelial cell division promote intra/extravasation of leukocytes? In a more clinically relevant aspect, could the modulation of mitotic cancer cells at the tumor’s border become a weak spot allowing immune cell infiltration? These and more questions will be waiting for an answer, until whenever some pioneers will be able to move forward, just like in this model.
